# Intelligent Parameter Identification for Robot Servo Controller Based on Improved Integration Method

**DOI:** 10.3390/s21124177

**Published:** 2021-06-18

**Authors:** Ye Li, Dazhi Wang, Shuai Zhou, Xian Wang

**Affiliations:** 1 School of Information Science and Engineering, Northeastern University, Shenyang 110819, China; 1710242@stu.neu.edu.cn (Y.L.); 1610234@stu.neu.edu.cn (S.Z.); 2 Northeast Branch of State Grid Corporation of China, No.1, Yingpan North Street, Hunnan District, Shenyang 110180, China; 1510307@stu.neu.edu.cn

**Keywords:** robot servo controller, moment of inertia, viscous friction coefficient, improved integration method, incremental probabilistic neural network, improved Gravitational Search Algorithm

## Abstract

With the rise of smart robots in the field of industrial automation, the motion control theory of the robot servo controller has become a research hotspot. The parameter mismatch of the controller will reduce the efficiency of the equipment and damage the equipment in serious cases. Compared to other parameters of servo controllers, the moment of inertia and friction viscous coefficient have a significant effect on the dynamic performance in motion control; furthermore, accurate real-time identification is essential for servo controller design. An improved integration method is proposed that increases the sampling period by redefining the update condition in this paper; it then expands the applied range of the classical method that is more suitable for the working characteristics of a robot servo controller and reducesthe speed quantization error generated by the encoder. Then, an optimization approach using the incremental probabilistic neural network with improved Gravitational Search Algorithm (IGSA-IPNN) is proposed to filter the speed error by a nonlinear process and provide more precise input for parameter identification. The identified inertia and friction coefficient areused for the PI parameter self-tuning of the speed loop. The experiments prove that the validity of the proposed method and, compared to the classical method, it is more accurate, stable and suitable for the robot servo controller.

## 1. Introduction

At present, industrial robots have played an increasingly important role in industrial production against the backdrop of global industrial manufacturing’s gradual intelligent development. In order to make the industrial robot capable of more complex work, the robot control technology requires to have the characteristics of high speed, high precision, multi-coordination and has good dynamic performance. In summary, finding a way to control industrial robotsin a way that makes them respond quickly to the predetermined trajectory instructions while maintaining a high dynamic tracking accuracy, aiming to achieve stable operation, has become an important research topic in the field of industrial robot motion control [1,2,3,4,5,6].

Selecting an appropriate controller is important for system design. Advanced controllers have been proposed recent studies. In [7], three controllers (PID, Stanley and Sliding Mode Control) weredesigned. Their performance was compared the performance in seven different environments. Their anti-disturbance properties showed which controllers performed well in each environment. In [8], the comprehensive performance of four controllers (linear quadratic regulator, model predictive controller, $H_{\infty }$ loop shaping and μ-synthesis) was compared based on position tracking, vibration damping and control effort for position control and mechanical vibration suppression in compliant link mechanism. The results showed that different controllers have different advantages for their main purposes. However, the classic proportion–integration–differentiation (PID) controller has the advantage of its simple structure, computational efficiency and low cost compared with other advanced controllers; therefore, it is the common controller for industrial robots.

The parameters of the servo controller such as moment of inertia and viscous friction coefficient are extremely important, as they affect the dynamic performance of the controller designs. For example, the inertia and friction coefficient are used to design the speed loop controller and, as the parameters increase, the dynamic responsiveness of the system will be reduced; as the parameters decrease, the system will oscillate. In addition, when the inertia and friction coefficient change which are used to design the position loop controller, the system response will be slow and overshoot [9]. Therefore, by identifying the inertia and friction coefficient online and self-tuning the parameters of the controller in different industrial application environments, it can have higher response speed and control precision so that the industrial robot system is more accurate and efficient.

In general, the conventional inertia and friction coefficient identification method can be divided into offline identification and online identification [10]. The offline identification needs to be in a certain reference frame that has fixed parameters and more applicable to the debugging process. The effectiveness of the offline identification method has been verified in the pertinent literature. In [11], the moment of inertia and friction torque coefficient wereobtained exactly and simultaneously from a half-period integration method using a very low-frequency sinusoidal speed control; it utilized the fact that the sinusoidal speed is in phase with the friction torque and out of phase with the inertia torque. Additionally, in [12], a method based on the addition of zero mean sinusoidal perturbation to the permanent magnet synchronous motor (PMSM) drive system was proposed in order to estimate the combined moment of inertia within one sinusoidal cycle of perturbation that did not need complex algorithm and the viscous friction can be eliminated. Finally, in [13], a load torque observer based on sliding mode was proposed for offline inertia identification which is applicable for the mismatch of the inertia always causes load torque observation error under dynamic conditions. However, because of the offline identification requires the drive system to have fixed inertia and friction coefficient, in practice, the parameters of the system will fluctuate with the change of the attitude of the industrial robot and have problems such as a long identification time, low precision and large storage space; therefore, it is not suitable for the dynamic adjustment and control of industrial robot [14,15].

Compared to the offline identification method, the online identification method does not need pre-estimate and modeling of the system that means the inertia and friction coefficient can be indeterminate, so it has fewer limitations and covers more complicated situations of the industrial robot. The commonly used online identification methods cover three categories: (1) establishment of high order observer or multi-algorithm compound structure; (2) use the integration method counteracts the effect of the uncertain load torque and periodically updates the observed inertia; (3) various kinds of optimized neural networks are used to identify and self-tune the real-time parameters of the drive system. In general, for the observer method, it has the advantages of strong anti-disturbance and robustness. In [16], a robust and stable disturbance observer using radial basis function network (RBFN) was proposed so that the parameter variation and external load disturbances can be approximated, and an additional robust control was introduced to compensate for the identification error so that the whole system is stable. In [17], in order to reduce the calculation burden of the parameters identification, a novel inertia identification algorithm based on the fixed-order empirical frequency-domain optimal parameter estimation was proposed which is more precise and more robust against system delay and errors. In [18], a single recursive least square estimation method with forgetting factor was proposed to estimate inertia for solving the identification problem that the simultaneous change of load torque and inertia, and the method avoids designing two separate observers and simplifies the computations. Additionally, the integration method can identify the inertia and friction coefficient smoothly and has a stronger anti-noise ability. In article [19], a variable period velocity differential calculation strategy was proposed to reduce the measurement noise caused by the quantization error of the encoder and the moment of inertia is updated in real time. The integration method can also be combined with the observers, as mentioned in article [20], which combines the extended state observer (ESO) with the integration method to design an adaptive controller for the PMSM speed-regulation system and improve the adaptation of the system to the variations of inertia. Finally, compared to the methods mentioned above, the neural network is more as an optimization tool to make the result of parameter identification even more excellent. In [21], a real-time moment of inertia, the identification technique using a Petriprobabilistic fuzzy neural network with an asymmetric membership function was proposed by combining the neural network and integral method to optimize the identification results for the servo drive system.

Although the methods mentioned above have been applied in mechatronic servo systems successfully, there are still some problems to be solved. The method of high order observer or multi-algorithm compound structure has the problems of transient observation result fluctuation and asymptotic convergence verification, and the neural network is more combined with other methods to optimize the identification results. Frequency response is also a reasonable and effective method for the identification of dynamic parameter; however, the signal is inevitably affected by various noises and affect the parameter identification accuracy of frequency response. At the same time, the frequency response is applicable to the low degree of freedom system with known parameters and complete modal information is required; otherwise, the eigenmatrix cannot be inverse in the loss case, so it is not suitable for the parameter identification of the industrial robot. By contrast, the integration method can improve the above problems by counteracting the influence of uncertain load torque and updating the inertia and friction coefficient periodically. However, the conventional integration method has the condition that the speed and acceleration need to be periodic to ensure the system can reach a steady state relatively, and the speed quantization error will cover the real speed change information. For industrial robots, the condition is too harsh, as the robots usually work at irregular speed or position work commands. To reduce the impact of these problems, an improved integration method is proposed to identify the moment of inertia and viscous friction coefficient of the industrial robot servo controller that increases the sampling period by redefining the update condition in this paper, so that the method is no longer limited to periodic speed and acceleration conditions. Then, an optimization approach using the IGSA-IPNN is proposed to filter the speed error which by adding hidden layer of neurons to improve the accuracy of traditional probabilistic neural network training until the training error meets the expectation. As opposed to adding filters to the system, the neural network can easily learn additional information from new samples that improve the identification accuracy as the robot runs and has strong robustness; the oscillation phenomenon of the filter at resonant frequency can also be avoided and the system complexity is simplified. The IGSA is used to optimize the threshold of neural network; the poor local optimization ability and premature convergence problems of the original method are solved and the output of the neural network is more precise.

This paper is organized as follows: Section 2 presents the classical integration method for the inertia and friction coefficient identification and the process of the controller self-tuning. Section 3 presents the improved integration method and the process of the IGSA-IPNN optimized method. Section 4 presents the simulation experiment results and the validity of the proposed method is verified. Section 5 concludes this paper.

## 2. Classical Integration Method of Moment of Inertia and Viscous Friction Coefficient Identification

In order to provide a context for an in-depth understanding of the identification method proposed and the residue problems with the classical method in this paper, the classical integration method and the controller self-tuning method are introduced in this part.

### 2.1. Mechanical System Model

The motion state of a robot servo controller motor is described by its mechanical motion equation and expressed as follows [22]:

(1) $$J_{m}\frac{d\omega_{m}}{dt}=T_{e}-T_{f}-T_{L}$$

(2) $$T_{e}=\frac{3}{2}P_{n}\cdot \left[\psi_{f}+\left(L_{d}-L_{q}\right)i_{d}\right]\cdot i_{q}$$

where $T_{e}$ and $T_{L}$ are the electrical torque and load torque of servo motor system, respectively. $\omega_{m}$ is the mechanical speed and $J_{m}$ is the moment of inertia. $T_{f}$ is the friction torque consisting of three types of friction which contains of static friction torque, coulomb friction torque and viscous friction. The friction torque equation can be given as:

(3) $$T_{f}=\left(T_{s}-T_{c}\right)e^{-\left(\omega_{m}/\omega_{s}\right)}\mathrm{sign}\left(\omega_{m}\right)+T_{c}\mathrm{sign}\left(\omega_{m}\right)+B_{m}\omega_{m}$$

where $T_{s}$ is the static friction torque, $T_{c}$ is the coulomb friction torque, $\omega_{s}$ is the Stribeck velocity parameter and $B_{m}$ is the viscous friction coefficient. Usually, the first two terms in Equation (3) are ignored when the motor is in normal operation because, compared with the viscous friction torque, the resistance to the motor operation of static friction and coulomb friction is weak. Inversely, as the only type of linear friction, the value of viscous friction torque is proportional to the rotating speed. Finally, the mechanical motion equation of a servo motor can be expressed as

(4) $$T_{e}=J_{m}\frac{d\omega_{m}}{dt}+B_{m}\omega_{m}+T_{L}$$

**Figure 1:**
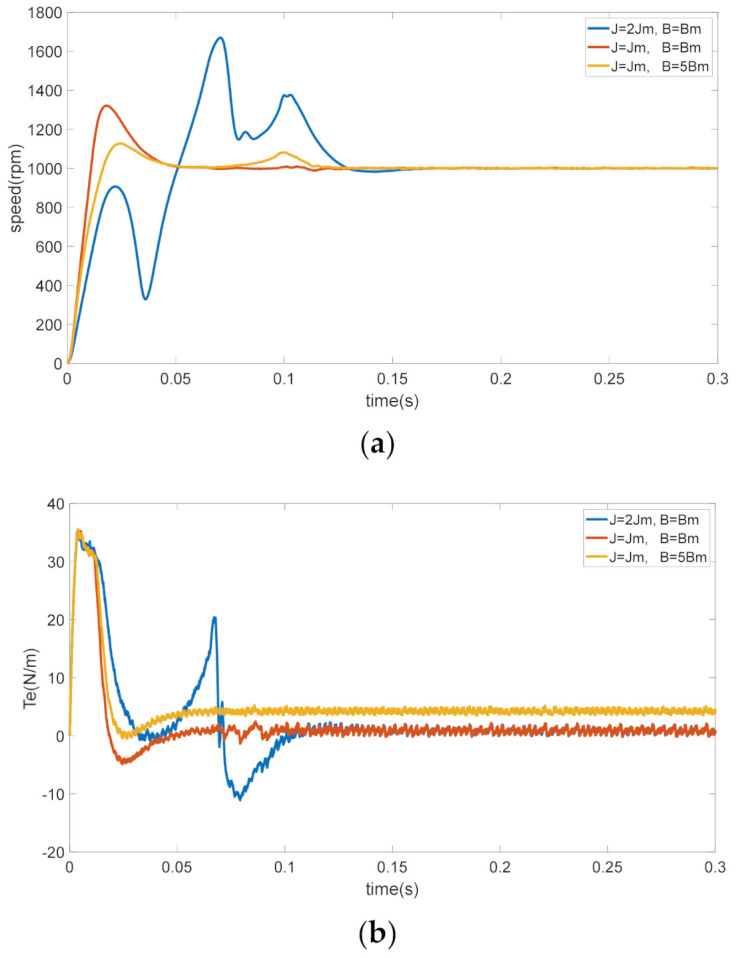
Comparisons in the case of $J=J_{m},\;J=2J_{m},\;B=5B_{m}$. (**a**) Speed response. (**b**) Torque response.

The dynamic performance of the servo system controller is sensitive to the variation of mechanical parameters; if there is a deviation value to the original moment of inertia and viscous friction coefficient of the servo motor system, the control performance of the controller will be degraded when the mechanical parameters of the controller are fixed. In order to prove this phenomenon, a simulation experiment is shown in Figure 1.

The red lines in Figure 1 show the response curves of speed and torque when the inertia of the system is $J_{m}$. The speed and torque response have a slight oscillate and a short settling time before stabilizing. The blue lines and yellow lines in Figure 1 show the response curves of speed and torque when the inertia of the system is doubled and the viscous friction coefficient is increased to 5$B_{m}$. From the curves, we can see that when the inertia is doubled, the speed and torque response have a violent oscillation and need a longer time to stabilize. Similarly, when the viscous friction coefficient is increased to five times, the speed and torque response need longer stabilization time, and the torque response will eventually have a deviation value (no-load).Except the moment of inertia and viscous friction coefficient, the other paraments of the system are the same.

From the simulation results, we can see that the control performance of the system will decline if the moment of inertia and viscous friction coefficient of the system vary largely. In order to ensure the stability of the system performance, is necessary to identify the inertia and friction to handle these kinds of changes.

### 2.2. Moment of Inertia Identification

To identify the moment of inertia, the integration method which was proposed by Awaya et al. in 1992 is adopted in this paper and the effect of uncertain load torque can be counteracted [23]. Assuming that the viscous friction coefficient and load torque are constant, the moment of inertia changes slowly in each identification period. The $\Delta J_{m}$ and ${\hat{J}}_{m}$ are deviation value and observe value of the inertia, respectively, and the $\Delta J_{m}$ is caused by a disturbance torque or by an estimate error. The mechanical motion equation can be rewritten as:

(5) $$T_{e}\left(t\right)={\hat{J}}_{m}\frac{d\omega_{m}\left(t\right)}{dt}+\Delta J_{m}\frac{d\omega_{m}\left(t\right)}{dt}+B_{m}\omega_{m}\left(t\right)+T_{L}$$

Multiplying the derivative of velocity on both sides, Equation (5) can be expressed as

(6) $$\begin{array}{l} T_{e}(t)\frac{d\omega_{m}(t)}{dt}={\hat{J}}_{m}{\left(\frac{d\omega_{m}(t)}{dt}\right)}^{2}+\Delta J_{m}{\left(\frac{d\omega_{m}(t)}{dt}\right)}^{2}\\ \;\;\;\;\;\;\;\;\;\;\;\;\;\;\;\;\;\;\;\;\;+B_{m}\omega_{m}(t)\frac{d\omega_{m}(t)}{dt}+T_{L}\frac{d\omega_{m}(t)}{dt} \end{array}$$

Then, integrating both sides of (6), the integration form of the mechanical motion equation can be expressed as

(7) $$\begin{array}{l} \int_{t_{i}}^{t_{f}}T_{e}(t)\frac{d\omega_{m}(t)}{dt}dt={\hat{J}}_{m}\int_{t_{i}}^{t_{f}}{\left(\frac{d\omega_{m}(t)}{dt}\right)}^{2}dt+\Delta J_{m}\int_{t_{i}}^{t_{f}}{\left(\frac{d\omega_{m}(t)}{dt}\right)}^{2}\\ \;\;\;\;\;\;\;\;\;\;\;\;\;\;\;\;\;\;\;\;\;\;\;\;\;\;\;\;\;\;\;\;\;\;\;\;+B_{m}\int_{t_{i}}^{t_{f}}\omega_{m}(t)\frac{d\omega_{m}(t)}{dt}dt+T_{L}\int_{t_{i}}^{t_{f}}\frac{d\omega_{m}(t)}{dt}dt \end{array}$$

where $t_{i}$ and $t_{f}$ are the initial time and finish time of the identification of cycle, respectively. By calculating the last two integral terms on the right-hand side of (7) and the expansion of the integral terms can be expressed as

(8) $$\left\{\begin{array}{l} B_{m}\int_{t_{i}}^{t_{f}}\omega_{m}(t)\frac{d\omega_{m}(t)}{dt}dt=\frac{1}{2}B_{m}\left[\omega_{m}{\left(t_{f}\right)}^{2}-\omega_{m}{\left(t_{i}\right)}^{2}\right]\\ T_{L}\int_{t_{i}}^{t_{f}}\frac{d\omega_{m}(t)}{dt}dt=T_{L}\left[\omega_{m}\left(t_{f}\right)-\omega_{m}\left(t_{i}\right)\right] \end{array}\right.$$

Substituting (8) to (7), the observe value and deviation value of the moment of inertia can be expressed as

(9) $$\begin{array}{l} {\hat{J}}_{m}+\Delta J_{m}=\frac{\int_{t_{i}}^{t_{f}}T_{e}(t)\frac{d\omega_{m}(t)}{dt}dt}{\int_{t_{i}}^{t_{f}}{\left(\frac{d\omega_{m}(t)}{dt}\right)}^{2}dt}-\frac{1}{2}B_{m}\frac{\left[\omega_{m}{\left(t_{f}\right)}^{2}-\omega_{m}{\left(t_{i}\right)}^{2}\right]}{\int_{t_{i}}^{t_{f}}{\left(\frac{d\omega_{m}(t)}{dt}\right)}^{2}dt}\\ \;\;\;\;\;\;\;\;\;\;\;\;\;\;\;\;\;\;\;\;\;-T_{L}\frac{\left[\omega_{m}\left(t_{f}\right)-\omega_{m}\left(t_{i}\right)\right]}{\int_{t_{i}}^{t_{f}}{\left(\frac{d\omega_{m}(t)}{dt}\right)}^{2}dt} \end{array}$$

According to Equation (9), since the numerator of the second and the third term is a constant value, if the identification period is sufficiently long, the denominator of the second and the third term will extremely large compared to the numerator that cause the whole term converge to zero and can be neglected. Nevertheless, a long period is not suitable for the real-time inertia identification. Hence, it is assumed that the speed at $t_{i}$ and $t_{f}$ is equal and the second and the third term can be set to zero. In order to satisfy this condition, a periodic speed formula can be expressed as

(10) $$\omega_{m}\left(t\right)=\omega_{m}\left(t+T_{pi}\right),\;\frac{d\omega_{m}}{dt}\neq 0$$

where the $T_{pi}$ is the inertia identification period and the second and the third term is equal to zero. The moment of inertia, including the observe value and deviation value by integration method, can be expressed as

(11) $${\hat{J}}_{m}+\Delta J_{m}=\frac{\int_{t}^{t+T_{pi}}T_{e}(t)\frac{d\omega_{m}(t)}{dt}dt}{\int_{t}^{t+T_{pi}}{\left(\frac{d\omega_{m}(t)}{dt}\right)}^{2}dt}$$

Eventually, the recursion formula of the identification inertia can be expressed as ($k\in N^{+}$):

(12) $$\begin{array}{l} {\hat{J}}_{m}\left(k+1\right)={\hat{J}}_{m}\left(k\right)+\Delta J_{m}\left(k\right)\\ \;\;\;\;\;\;\;\;\;\;\;\;\;\;=\frac{\int_{kT_{pi}}^{\left(k+1\right)T_{pi}}T_{e}(t)\frac{d\omega_{m}(t)}{dt}dt}{\int_{kT_{pi}}^{\left(k+1\right)T_{pi}}{\left(\frac{d\omega_{m}(t)}{dt}\right)}^{2}dt} \end{array}$$

For the purpose of proving the convergence of the recursion Formula (12), assuming the total deviation value in the identification period is $\Delta J_{total}$, it can be expressed as

(13) $$\underset{k\to \infty }{\lim}\Delta J_{total}=\Delta J_{m}\left(k\right)$$

then,

(14) $$J_{m}=\underset{k\to \infty }{\lim}{\hat{J}}_{m}\left(k\right)$$

From Equation (14), it can be seen that the estimated moment of inertia converges to a constant and proves the convergence of the integration method.

### 2.3. Viscous Friction Coefficient Identification

Similar to the identification of the moment of inertia, the identification of viscous friction coefficient using the integration method also needs the mechanical motion equation. First, taking the derivative of each term, then multiplying the derivative of velocity, Equation (5) can be transformed:

(15) $$\begin{array}{l} \frac{dT_{e}(t)}{dt}\cdot \frac{d\omega_{m}(t)}{dt}=J_{m}\cdot \frac{d^{2}\omega_{m}(t)}{dt^{2}}\cdot \frac{d\omega_{m}(t)}{dt}+{\hat{B}}_{m}\cdot \int_{t_{i}}^{t_{f}}{\left(\frac{d\omega_{m}(t)}{dt}\right)}^{2}\\ \;\;\;\;\;\;\;\;\;\;\;\;\;\;\;\;\;\;\;\;\;\;\;\;\;\;\;\;+\Delta B_{m}\cdot \int_{t_{i}}^{t_{f}}{\left(\frac{d\omega_{m}(t)}{dt}\right)}^{2}+\frac{dT_{L}}{dt}\cdot \frac{d\omega_{m}(t)}{dt} \end{array}$$

where the $\Delta B_{m}$ and ${\hat{B}}_{m}$ are the deviation value and observe value of the viscous friction coefficient. Integrating both sides of (15), the integration form of the mechanical motion equation can be expressed as

(16) $$\begin{array}{l} \int_{t_{i}}^{t_{f}}\frac{dT_{e}(t)}{dt}\cdot \frac{d\omega_{m}(t)}{dt}=J_{m}\cdot \int_{t_{i}}^{t_{f}}\frac{d^{2}\omega_{m}(t)}{dt^{2}}\cdot \frac{d\omega_{m}(t)}{dt}+{\hat{B}}_{m}\cdot \int_{t_{i}}^{t_{f}}{\left(\frac{d\omega_{m}(t)}{dt}\right)}^{2}\\ \;\;\;\;\;\;\;\;\;\;\;\;\;\;\;\;\;\;\;\;\;\;\;\;\;\;\;\;\;\;\;\;\;\;\;\;\;\;+\Delta B_{m}\cdot \int_{t_{i}}^{t_{f}}{\left(\frac{d\omega_{m}(t)}{dt}\right)}^{2}+\int_{t_{i}}^{t_{f}}\frac{dT_{L}}{dt}\cdot \frac{d\omega_{m}(t)}{dt} \end{array}$$

Assuming that the load torque is a constant value or varies slowly during the identification period, the derivative of the load torque can be approximated to zero. When calculating the first and third terms on the right-hand side of (16), the expansion of the integral terms can be expressed as

(17) $$\left\{\begin{array}{l} J_{m}\cdot \int_{t_{i}}^{t_{f}}\frac{d^{2}\omega_{m}(t)}{dt^{2}}\cdot \frac{d\omega_{m}(t)}{dt}dt\\ =\frac{1}{2}J_{m}\cdot \left[{\left(\frac{d\omega_{m}\left(t_{f}\right)}{dt}\right)}^{2}-{\left(\frac{d\omega_{m}\left(t_{i}\right)}{dt}\right)}^{2}\right]\\ \frac{dT_{L}}{dt}≅0,\int_{t_{i}}^{t_{f}}\frac{dT_{L}}{dt}\cdot \frac{d\omega_{m}(t)}{dt}dt=0 \end{array}\right.$$

Substituting (17) with (16), the viscous friction coefficient can be expressed as

(18) $$\begin{array}{l} {\hat{B}}_{m}+\Delta B_{m}=\frac{\int_{t_{i}}^{t_{f}}\frac{dT_{e}(t)}{dt}\cdot \frac{d\omega_{m}(t)}{dt}dt}{\int_{t_{i}}^{t_{f}}{\left(\frac{d\omega_{m}(t)}{dt}\right)}^{2}dt}\\ \;\;\;\;\;\;\;\;\;\;\;\;\;\;\;\;\;\;\;\;-\frac{1}{2}J_{m}\cdot \frac{\left[{\left(\frac{d\omega_{m}\left(t_{f}\right)}{dt}\right)}^{2}-{\left(\frac{d\omega_{m}\left(t_{i}\right)}{dt}\right)}^{2}\right]}{\int_{t_{i}}^{t_{f}}{\left(\frac{d\omega_{m}(t)}{dt}\right)}^{2}dt} \end{array}$$

According to Equation (18), similar with the previous analysis, the second term can also be neglected if the identification period is sufficiently long. In order to adapt to the real-time identification method in this paper, assuming the absolute value of accelerated speed at $t_{i}$ and $t_{f}$ is equal or close to each other, the second term can be set to zero. In order to satisfy this condition, a periodic accelerated speed formula can be expressed as

(19) $$\frac{d\omega_{m}\left(t\right)}{dt}=\frac{d\omega_{m}\left(t+T_{pf}\right)}{dt},\frac{d\omega_{m}\left(t\right)}{dt}\neq 0$$

where the $T_{pf}$ is identification period of the viscous friction coefficient, it can be expressed as

(20) $${\hat{B}}_{m}+\Delta B_{m}=\frac{\int_{t}^{t+T_{pf}}\frac{dT_{e}(t)}{dt}\cdot \frac{d\omega_{m}(t)}{dt}dt}{\int_{t}^{t+T_{pf}}{\left(\frac{d\omega_{m}(t)}{dt}\right)}^{2}dt}$$

Similar with the inertia, the recursion formula of the identification inertia can be expressed as

(21) $$\begin{array}{l} {\hat{B}}_{m}\left(k+1\right)={\hat{B}}_{m}\left(k\right)+\Delta B_{m}\left(k\right)\\ \;\;\;\;\;\;\;\;\;\;\;\;\;=\frac{\int_{kT_{pf}}^{\left(k+1\right)T_{pf}}\frac{dT_{e}(t)}{dt}\cdot \frac{d\omega_{m}(t)}{dt}dt}{\int_{kT_{pf}}^{\left(k+1\right)T_{pf}}{\left(\frac{d\omega_{m}(t)}{dt}\right)}^{2}dt} \end{array}$$

The convergence of the integration method can be proved by the following formula:

(22) $$\left\{\begin{array}{l} \underset{k\to \infty }{\lim}\Delta B_{total}=\Delta B_{m}\left(k\right)\\ B_{m}=\underset{k\to \infty }{\lim}{\hat{B}}_{m}\left(k\right) \end{array}\right.$$

The moment of inertia and viscous friction coefficient can be used for adjustment of the gains of controller and observer. In this paper, the parameters are directed at tuning the dynamics proportional gain and integral gain of the speed loop controller.

### 2.4. Speed Loop Controller Parameter Auto-Tuning

After identification of the inertia and viscous friction coefficient, the speed controller parameter can be calculated to improve the dynamic performance of the system. Considering the negligible overshoot in a step tracking response, good regulating characteristics and zero steady-state error, the proportion integration (PI) controller is used for speed control in this paper. The transfer function of rotor speed response to command input can be expressed as

(23) $$\begin{array}{l} \frac{\omega_{m}}{\omega_{m}^{*}}=\frac{K_{I}K_{t}}{J_{m}s^{2}+\left(B_{m}+K_{P}K_{t}\right)s+K_{I}K_{t}}\\ \;\;\;\;\;\;≅\frac{\omega_{n}^{2}}{s^{2}+2\xi \omega_{n}s+\omega_{n}^{2}} \end{array}$$

where $K_{P}$ and $K_{I}$ represent proportional and integral gain, respectively. $\omega_{n}$ is the natural frequency and ξ is the system damping ratio, the relationship between them can be expressed as

(24) $$\xi =\frac{B_{m}+K_{P}K_{t}}{2{\left(J_{m}\cdot K_{I}K_{t}\right)}^{1/2}}$$

(25) $$\omega_{n}={\left(\frac{K_{I}K_{t}}{J_{m}}\right)}^{1/2}$$

The overshoot of the step function response of (23) is prevented by setting the damping ratio to 1 just that the absence of zeros and then the unit step response can be expressed as

(26) $$\omega_{m}\left(t\right)=1-e^{-\omega_{n}t}\left(1+\omega_{n}t\right)$$

Usually, the response time of the unit step response is defined as the time rise from 0 to 90% of its final value for convenience of designing the PI controller quantitatively. Equation (26) can be rewritten as:

(27) $$0.9=1-e^{-\omega_{n}t_{re}}\left(1+\omega_{n}t_{re}\right)$$

where $t_{re}$ is the response time, solve the above equation to obtain $\omega_{n}$ and then the parameters of PI controller can be expressed as

(28) $$K_{p}=\frac{2J_{m}\omega_{n}-B_{m}}{K_{t}}\;\;K_{I}=\frac{J_{m}\omega_{n}^{2}}{K_{t}}$$

**Figure 2:**
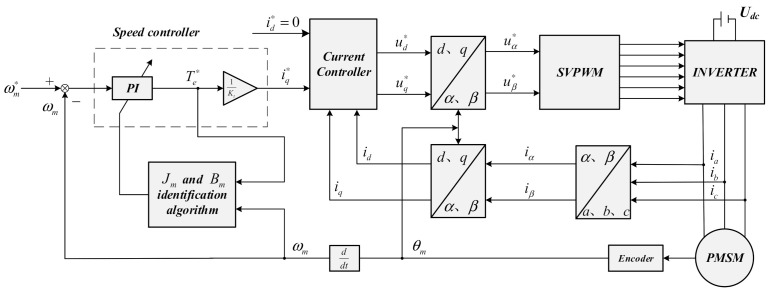
Block diagram of the PMSM drive system parameters identification.

From the above formula, the estimated moment of inertia and viscous friction coefficient are substituted into (28) to adjust the gain of the PI speed controller to accomplish the parameters auto-tuning of the PMSM servo drive system and improve the dynamic performance of the system at different running conditions. The block diagram of the PMSM drive system parameters identification is shown in Figure 2.

## 3. Improved Integration Identification Method and IPNN-IGSA Optimization Method

According to the above analyses, the classical identification process of the moment of inertia and viscous friction coefficient has been introduced. However, the classical method still has some weakness that causes the applied range of it under restrictions. In general, the limitations of the classical method include the following:

1. The most important condition of the conventional integration method is that the speed and accelerated speed need to be periodic to ensure the system reach a steady state relatively, meaning that the sampling period is fixed. However, in fact, this condition is too rigorous, because the commands of the servomotor equipment are irregular under normal circumstances.
2. Several key parameters in the conventional integration method have preconditions. For example, the load torque $T_{L}$ needs to be a constant value or vary slowly during the identification period. Similarly, the moment of inertia $J_{m}$ and viscous friction coefficient $B_{m}$ also need to be constant or vary slowly. The motor torque constant $K_{t}$ needs to be accurate.
3. Although the conventional integration method has ability of noise reduction to decrease the quantization error from encoder, if the accelerated speed changes slowly, the quantization error will cover the real speed change information and make the system instability.

For the purpose of expanding the applied range of the conventional integration method to limit the negative impacts, an improved integration method is proposed to weaken or avoid the first and third limitations, as mentioned above. The first limitation—i.e., the fixed sampling period of the conventional integration method being too rigorous and the accelerated speed measurement error being inversely related to the magnitude of the absolute value of the acceleration, leading to difficulties estimating the accelerated speed accurately when it is not obvious—causes the system to fail to provide a strict accelerated speed for inertia and friction coefficient identification. The third limitation—i.e., that since the quantization error from the encoder exists, the real speed feedback signals will contain noise signals—leads to the system being unable to provide accurate speed information for the identification of the inertia and friction coefficient.

In conclusion, to reduce the accelerated speed estimation error significantly and expand the applied range, an improved integration method by increasing sampling period for the process of the moment of inertia and viscous friction coefficient identification is proposed in this section. Meanwhile, to optimize the speed differential information and reduce the speed quantization error from the encoder, a smoothing approach using the IPNN-IGSA for the speed error measurement to filter out the information that the speed measurement error exceeds the error threshold is proposed in this section.

### 3.1. Improved Integration Identification Method for Inertia and Friction Coefficient

Most servomotor equipment works in position regulation mode; however the periodic speed command is not suitable for the position regulation mode. Inversely, frequent start–stop motion and reciprocating motion are more usual in position regulation mode. The characteristic of this motion is that the zero-speed point is frequently occurring and the accelerated speed during a start process and stop process is equal to each other. Therefore, readjust the sampling period of the moment of inertia from fixed period to speed equal to zero, readjust the sampling period of the viscous friction coefficient from fixed period to accelerated speed is different of the previous sampling period. The proposed update sampling period can be expressed by mathematical formula as:

(29) $$\left\{\begin{array}{l} \omega_{m}\left(k\right)=\omega_{m}\left(k-i\right)=0\\ \left|{d\omega_{m}\left(k\right)/dt}_{k}\right|\neq \left|{d\omega_{m}\left(k-i\right)/dt}_{k-i}\right| \end{array}\right.\;\frac{d\omega_{m}}{dt}\neq 0\;$$

**Figure 3:**
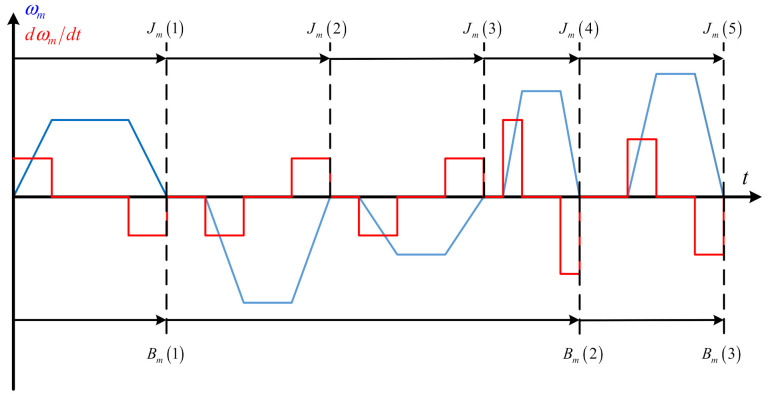
Schematic diagram of the proposed update sampling period.

Figure 3 is the schematic diagram of the proposed update sampling period. Compared with the sampling period rule of the conventional method, the proposed method in this paper has longer sampling period while reducing the accelerated speed estimation error and more suitable for the servo system position regulation mode. In order to get a better understanding of the proposed method, the detailed steps are mentioned as follows:

Updating sampling period of moment of inertia $J_{m}$: Preset a speed threshold $\omega_{th}$ and a time threshold $T_{th}$ firstly that the $\omega_{th}$ is the minimum speed that the system can identify and $T_{th}$ is the minimum identification duration time of the system. If the real-time speed of motor is larger than $\omega_{th}$ while the time of duration is larger than $T_{th}$, move to the next step, otherwise, repeat the above process. Then, update the $J_{m}$ if the motor speed $\omega_{m}$ is equal to zero, otherwise repeat the process. Finally, move back to the initial step to update the moment of inertia for the next period.

**Figure 4:**
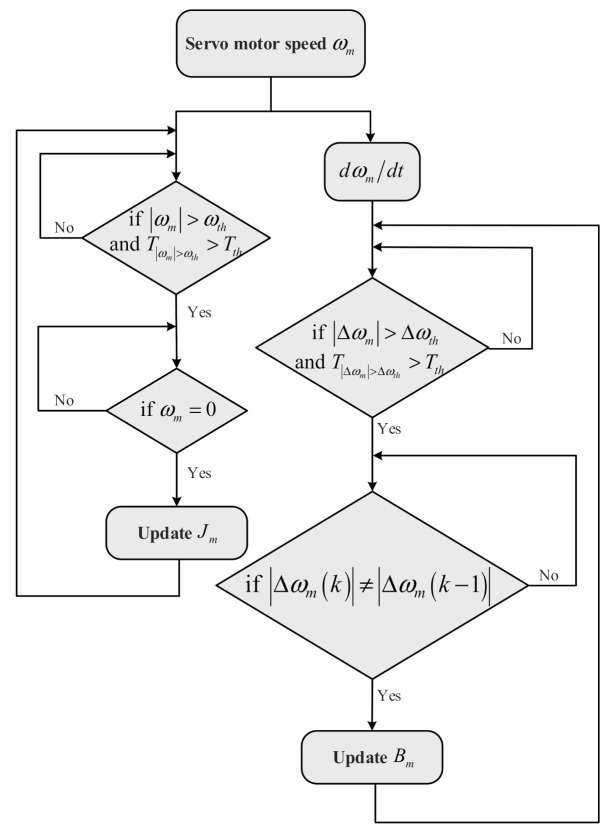
The flow chart of the proposed update process.

Updating sampling period of viscous friction coefficient $B_{m}$: Differentiating the speed firstly and preset the accelerated speed threshold $\Delta \omega_{th}$ which is the minimum accelerated speed that the system can identify. If the absolute value of the real-time accelerated speed is larger than $\Delta \omega_{th}$ while the time of duration is larger than time threshold $T_{th}$, move to the next step, otherwise, repeat the above process. Then, updating the $B_{m}$ if the absolute value of the real time accelerated speed $\left|\Delta \omega_{m}\left(k\right)\right|$ is not equal to the last absolute value of the accelerated speed $\left|\Delta \omega_{m}\left(k-1\right)\right|$, otherwise repeat the process. Finally, move back to the initial step to update the viscous friction coefficient for the next period. The flow chart of the update process is shown in Figure 4.

In conclusion, compared with the conventional method by fixed sampling period, the proposed method in this paper which increase the sampling period expand the applied range especially for the servo system position regulation mode and reduce the accelerated speed estimation error to provide a cleaner input for identification. The experimental verification part will be given below.

### 3.2. IPNN-IGSA Optimization Method for Speed Measurement Error

The network structure of the proposed IPNN-IGSA comprises four layers including the input layer, the pattern layer, the summation layer and the decision layer. In order to improve the accuracy of neural network training, a hidden layer of neurons is added between the input layer and the pattern layer until the training error meets the expectation. The signal propagation of each layer is described in detail as follows.

Input layer: The input data is derived from the training sample values, and then the input values are passed to all the pattern units. In this paper, the input data is the speed error e(t) which is obtained by subtracting the output of the estimated speed from the output of the command speed that can be expressed as

(30) $$e(t)=\omega_{m}^{*}-\omega_{m}$$

Suppose the output of the neural network is P types of data (in this paper, the neural network outputs two types of data, one is the data whose error is less than the threshold and can be used for the parameter identification, the other one is the data with larger errors) and the $Q_{p}$ is the neuron belonging to the p-th (p=1, 2) class. The scalar product formula is obtained by multiplying the speed error with the weighting coefficient $w_{p,q}$, and then input to the pattern layer for the next calculation. The scalar product is expressed as

(31) $$Z_{p,q}=e(t)\cdot w_{p,q}$$

Pattern layer: The number of pattern neurons is equal to the number of training data and each pattern neuron belongs to one type. The nonlinear operation is performed and use it as an activation function that is shown in (32). The probability of the output of the q neuron of type p is shown in (33).

(32) $$y_{p,q}=\exp\left[\left(Z_{p,q}-1\right)/\sigma^{2}\right]$$

(33) Φp,q(e)=1(2π)L2σL⋅yp,q

where σ is the smoothing parameter, and L is the dimension of training dataset.

Summation layer: This layer calculates the average value of the output of neurons in each pattern layer with the same type, and calculates the maximum probability that the data belongs to this category. The probability density function of the type p is obtained by Parzen window method, as shown in (34). Additionally, the output of the pth summation neuron is calculated by (35).

(34) $$f_{i}=\frac{\sum_{q=1}^{Q_{i}}\Phi_{p,q}(e)}{Q_{p}}$$

(35) $$S_{p}=\frac{1}{Q_{p}}\cdot \sum_{q=1}^{Q_{p}}\cdot \Phi_{p,q}(e)$$

Decision layer: The decision layer will estimate the probability of input vectors according to various types and select the neurons with the maximum probability density as the output. The output has only one weight, determined by the lost parameter, the prior probability, and the training pattern for each category. The output of the decision neuron is calculated by:

(36) $$e_{out}=\arg\max\left\{\frac{1}{2}S_{p}\right\}$$

**Figure 5:**
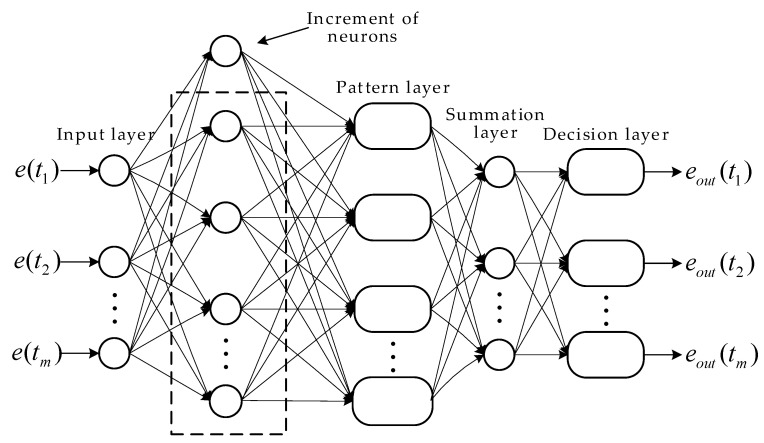
The network structure of IPNN.

In order to improve the accuracy of neural network filtering, the probabilistic neural network is trained by increasing the number of hidden layer neurons. When the training error does not meet the expectation, a hidden layer of neurons is added between the input layer and pattern layer until the training error meets the expectation. The network structure is shown in Figure 5.

For the neural network, the thresholds and weights are the most important parameters which determine the accuracy of the output results. In order to improve the problem, an intelligence optimization algorithm IGSA is applied in this paper to optimize the thresholds and weights.

There are four variables that are active gravitational mass, passive gravitational mass, inertial mass and position of each agent in IGSA optimization algorithm. Assuming that the system has N agents in the search area. Define the $X_{i}=\left(x_{i}^{1},\cdots x_{i}^{d},\cdots x_{i}^{N}\right)$ (i=1,2,⋯N) as the position of the i-th agent where $x_{i}^{d}$ is the d-th dimension value of the agent i. The force acted on agent i by agent j is shown in formula (37).

(37) $$F_{i,j}^{d}\left(t\right)=G\left(t\right)\frac{M_{pi}\left(t\right)\cdot M_{aj}\left(t\right)}{R_{i,j}\left(t\right)+\varepsilon }\left[x_{j}^{d}\left(t\right)-x_{i}^{d}\left(t\right)\right]$$

where $G\left(t\right)$ is gravitational coefficient which control the accuracy of the search and be decreased with the time passed by, $M_{pi}\left(t\right)$ is the passive gravitational mass connected with agent i, $M_{aj}\left(t\right)$ is the active gravitational mass connected with agent j, $R_{i,j}\left(t\right)$ is Euclidean distance between agent i and agent j, ε is a small constant.

The gravitational constant $G\left(t\right)$ is defined as:

(38) $$G\left(t\right)=G_{0}e^{-\tau \frac{t}{t_{\max}}}$$

where $G_{0}$ is the initial value, τ is the descending coefficient and $t_{\max}$ is the maximum number of the iterations.

The resultant force applied on agent i in d-th dimension is shown as

(39) $$F_{i}^{d}\left(t\right)=\underset{j\in K_{best}}{\Sigma }rand_{j}F_{i,j}^{d}\left(t\right)$$

The $K_{best}$ is the set of the first K agents with the biggest mass and will linearly decreased with iteration t. The $rand_{j}$ is a random variable in the interval $\left[0,1\right]$. According to the Newton’s second law, the acceleration of agent i in direction of the d-th dimension is as follows:

(40) $$a_{i}^{d}\left(t\right)=\frac{F_{i}^{d}\left(t\right)}{M_{i}\left(t\right)}$$

where $M_{i}\left(t\right)$ is the inertial mass of agent i. The inertia masses and gravitational are updated by the following equations.

(41) $$m_{i}\left(t\right)=\frac{fit_{i}\left(t\right)-f_{worst}\left(t\right)}{f_{best}\left(t\right)-f_{worst}\left(t\right)}$$

(42) $$M_{i}\left(t\right)=\frac{m_{i}\left(t\right)}{\overset{N}{\underset{j=1}{\Sigma }}m_{j}\left(t\right)}$$

The $fit_{i}\left(t\right)$ is the fitness value of agent i, $f_{best}\left(t\right)$ and $f_{worst}\left(t\right)$ are the best and worst fitness values, respectively.

**Figure 6:**
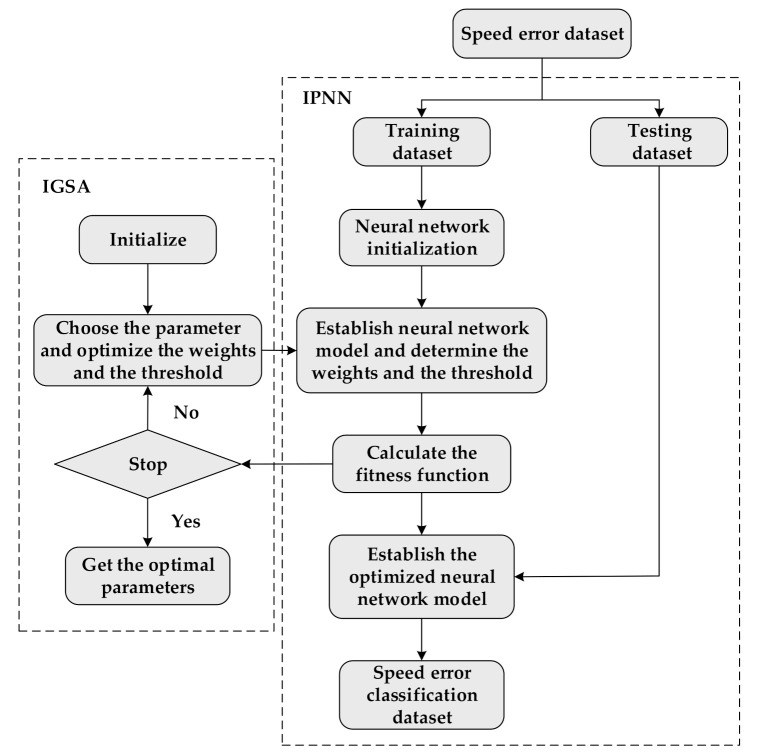
The flow chart of the IPNN-IGSA.

For the propose of improving the poor local optimization ability and premature convergence, IGSA adjusts the inertia weight and boundary variation dynamically. Finally, the speed and position of agent can be updated as follows:

(43) $$v_{i}^{d}\left(t+1\right)=rand_{i}\cdot w\left(t\right)\cdot v_{i}^{d}\left(t\right)+a_{i}^{d}\left(t\right)$$

(44) $$6x_{i}^{d}\left(t+1\right)=x_{i}^{d}\left(t\right)+v_{i}^{d}\left(t+1\right)$$

(45) $$w\left(t\right)=w_{\max}-\frac{w_{\max}-w_{\min}}{T}\cdot t$$

where $rand_{i}$ is a uniform random variable in the interval $\left[0,1\right]$, the size of $w_{\max}$ and $w_{\min}$ is according to the actual problem, the T is the maximum number of iterations. The flow chart of the IPNN-IGSA is shown in Figure 6.

## 4. Simulation Results and Discussion

To evaluate the performance of proposed improved integration method and IGSA-IPNN optimized method, a simulation experiment model for servo controller is built. The detailed parameters of the servo drive system are descripted in Table 1.

### 4.1. Performance of the Improved Integration Method

**Figure 7:**
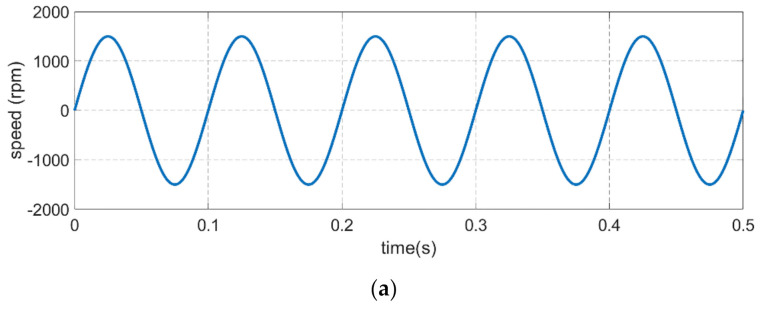

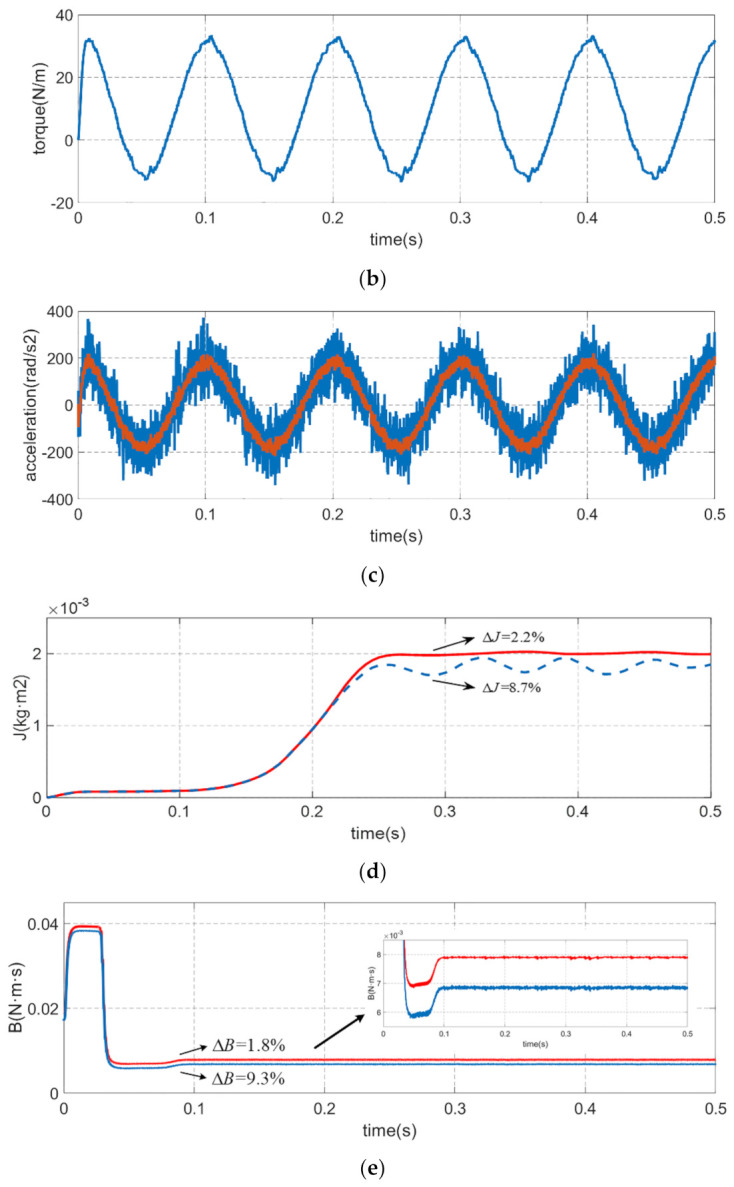
Comparison of inertia and friction coefficient identification results at the sinusoidal reference speed of 10 Hz, 1500 r/min which the blue line represents the classical integration method and red line represents the improved integration method. (**a**) Motor speed. (**b**) Electrical torque. (**c**) Acceleration. (**d**) Moment of inertia. (**e**) Viscous friction coefficient.

To validate the effectiveness of the proposed method, the sinusoidal reference speeds were chosen to be 10 Hz, 1500 r/min and 20 Hz, 3000 r/min which have been tested in the simulation experiment and the period is 0.5 s. Motor speed, electrical torque, acceleration, moment of inertia and viscous friction coefficient are the observation data of the simulation experiment. Figure 7 is the inertia and friction coefficient identification simulation experiment results of the classical fixed period integration method and the proposed improved update sampling period integration method and both methods are running at the sinusoidal reference speed of 10 Hz, 1500 r/min simultaneously. In Figure 7, except the speed and torque, the blue lines represent the classical fixed period integration method and the red lines represent the improved update sampling period integration method. From the simulation experiment data, the identification error of classical integration method is 8.7% while the identification error of improved integration method is 2.2% for the moment of inertia, and the identification error of classical integration method is 9.3% while the identification error of improved integration method is 1.8% for the viscous friction coefficient. Although the inertia and friction coefficient identification results of the improved update sampling period integration method still have a slight error in comparison with the actual value, compare to the classical fixed period integration method, the higher precision and stability demonstrate the excellent identification performance of the proposed method.

**Figure 8:**
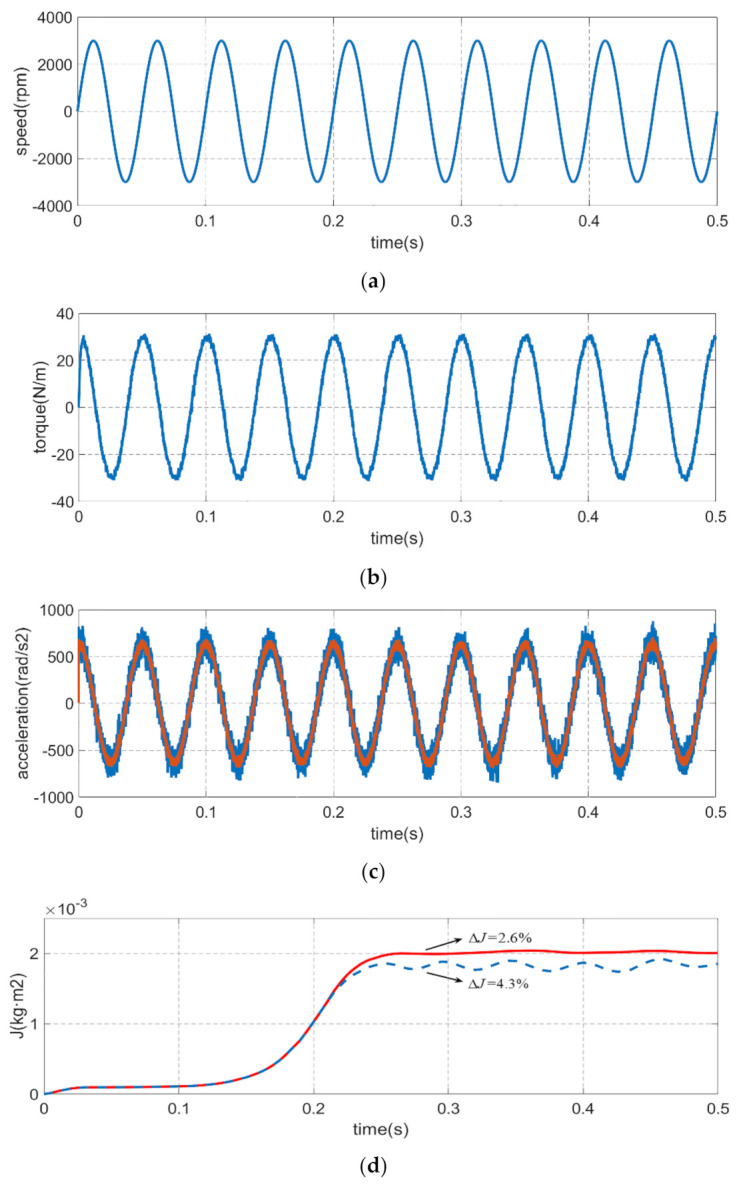

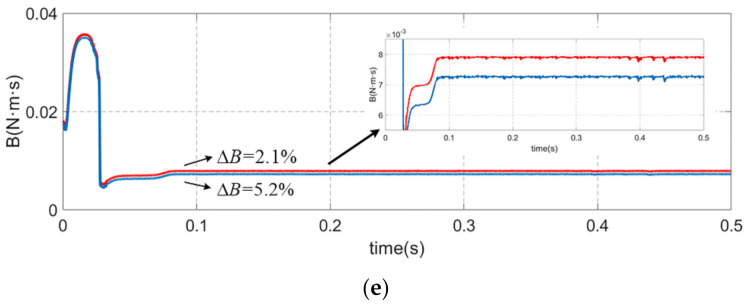
Comparison of inertia and friction coefficient identification results at the sinusoidal reference speed of 20 Hz, 3000 r/min which the blue line represents the classical integration method and red line represents the improved integration method. (**a**) motor speed. (**b**) electrical torque. (**c**) acceleration. (**d**) moment of inertia. (**e**) viscous friction coefficient.

Similarly, Figure 8 is the inertia and friction coefficient identification simulation experiment results at the sinusoidal reference speed of 20 Hz, 3000 r/min. The identification error of classical integration method is 4.3% while the identification error of improved integration method is 2.6% for the moment of inertia, and the identification error of classical integration method is 5.2% while the identification error of improved integration method is 2.1% for the viscous friction coefficient. The results also proved that the higher precision and stability of the proposed method compared to the classical integration method. Regardless of the speed and frequency of the servo motor, the inertia and friction coefficient identification accuracy of the method which proposed in this paper is superior to the classical method.

### 4.2. Performance of the Improved Integration Method combine with IGSA-IPNN

**Figure 9:**
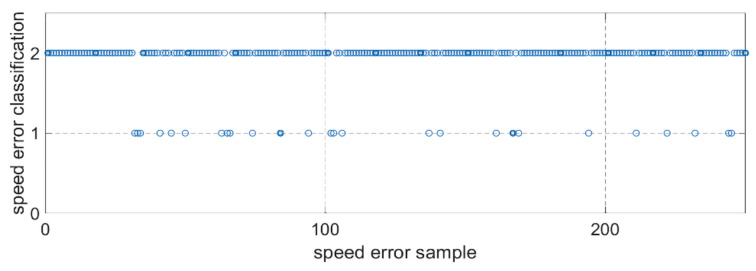
Part of the speed sample classification results by IGSA-IPNN.

**Figure 10:**
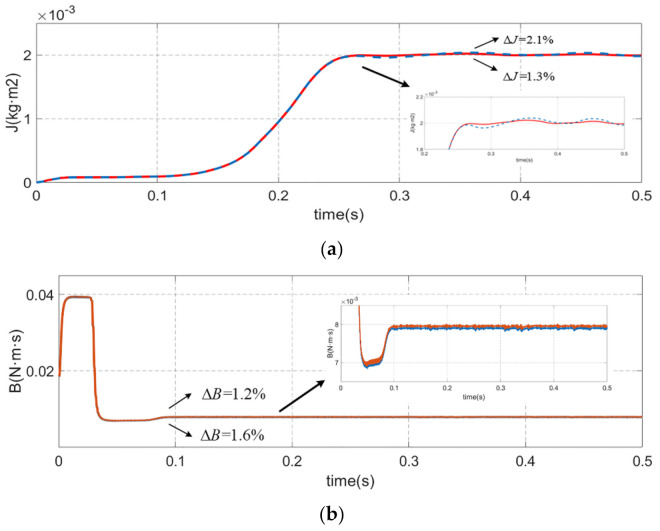
Comparison of inertia and friction coefficient identification results at the sinusoidal reference speed of 10 Hz, 1500 r/min which the blue line represents the single improved integration method and red line represents the improved integration method combine with IGSA-IPNN. (**a**) moment of inertia. (**b**) viscous friction coefficient.

The simulation experimental results of the proposed IGSA-IPNN to filter out the speed information with overlarge error at sinusoidal reference speed of 1500 r/min are shown in Figure 9 and Figure 10. Figure 9 is the part of the speed sample classification results by IGSA-IPNN, the classification 1 is the invalid speed sample which have excessive error and the classification 2 is the valid speed sample which the error is within acceptable limits and can be used for the inertia and friction coefficient identification. Figure 10 is the identification results of the inertia and friction coefficient; the red lines represent the improved update sampling period integration method combine with the IGSA-IPNN and the blue lines represent the improved update sampling period integration method.The identification error of the single improved integration method is 2.1%, while the identification error of improved integration method with IGSA-IPNN is 1.3% for the moment of inertia, and the identification error of single improved integration method is 1.6% while the identification error of improved integration method with IGSA-IPNN is 1.2% for the viscous friction coefficient. Thesimulation experiment show that the precision and stability of the identification results combined with the IGSA-IPNN are slightly better than the single method and the error is smaller. The validity of the proposed method is proved.

### 4.3. Stability Analysis of the Proposed Method

**Figure 11:**
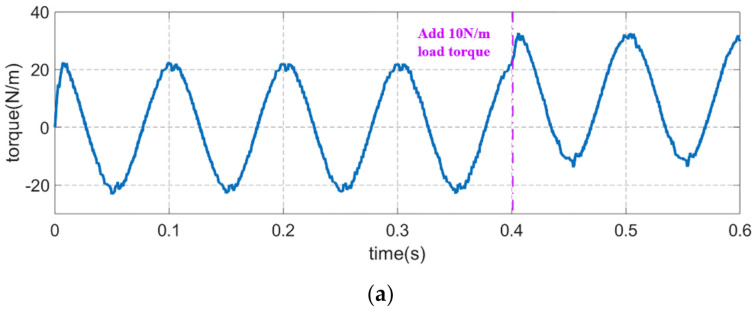

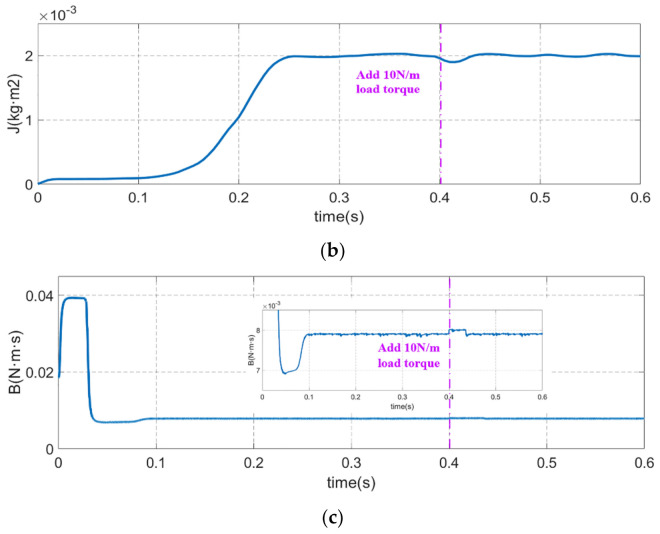
The anti-disturbance performance of the inertia and friction coefficient identification results: (**a**) Motor speed. (**b**) Moment of inertia. (**c**) Viscous friction coefficient.

In order to verify the stability of the proposed method, the stability analysis simulation experiment is carried out in this paper. A constant 10 N·m load torque is added at 0.4 s while the sinusoidal reference speed is 1500 r/min; the experiment results are shown in Figure 11. As can be seen from the experimental results, although the load torque disturbance causes a fluctuation of inertia and friction coefficient identification slightly, the result is ultimately restored to the exact value after a few tenths of a second. The anti-disturbance performance of the proposed method is proved.

## 5. Conclusions

In this paper, because the classical integration method has constraints that are too strict—which is not suitable for industrial robots, the speed quantization error generated by the encoder in the moment of inertia and viscous friction coefficient identification process—an improved integration method, combined with IGSA-IPNN, is proposed. First, as the industrial robot is difficult to satisfy the traditional methods of periodic speed and acceleration requirements when it work in speed or position control mode, an improved integral method by re-adjusting the update sampling period is proposed that make it stable and accurate in more a complex working environment and improve dynamic performance. In addition, an optimized neural network called IGSA-IPNN is proposed to filter out the speed differential information with error and provide precise input information for parameter identification in this paper. Finally, through different types of simulation experiment results and data analysis, the effectiveness and stability of the proposed method are verified. The experiments of parameter identification of industrial robot will be carried out in following research;the compensation of the moment of inertia, viscous friction coefficient and how to use the identification result to compensate the electromagnetic torque to eliminate the rotational speed error caused by are the future research priorities.

## Figures and Tables

**Table 1:** Main parameters of the servo controller model.

Parameter	Value
Rated power	600 W
Rated torque	1.91 N·m
Rated current	3.5 A
DC link voltage	220 V
Pole pairs	4
Stator resistance	0.643 Ω
d-axis inductance	5.25 mH
q-axis inductance	12 mH
Flux linkage	0.175 Wb
Inertia of servo motor	2 × 10^−3^ kg·m^2^
Viscous friction coefficient of servo motor	8 × 10^−3^ N·m·s
